# One-year trajectory of psychological symptoms in families of out-of-hospital cardiac arrest patients

**DOI:** 10.1186/s13054-025-05643-w

**Published:** 2025-10-24

**Authors:** Kasumi Shirasaki, Yohei Okada, Katsuhiro Horie, Tasuku Hada, Yu Watanabe, Nozomi Toya, Shutaro Isokawa, Toru Hifumi, Masaki Okajima, Norio Otani

**Affiliations:** 1 Department of Emergency and Critical Care Medicine, St. Luke’s International Hospital, 9-1 Akashicho, Chuo-Ku, Tokyo, 104-8560 Japan; 2 Department of Emergency and Disaster Medicine, Kanazawa University Hospital, 13-1 Takara-Machi, Kanazawa, 920-8640 Japan; 3 Prehospital and Emergency Research Centre, Health Services Research and Population Health, Duke-NUS Medical School, 8 College Rd, Singapore, 16985 Singapore; 4 Department of Preventive Services, Kyoto University, Yoshida Konoe-Cho, Sakyo-Ku, Kyoto, 606-8501 Japan; 5 Department of Emergency and General Medicine, Kyorin University School of Medicine, 6-20-2 Shinkawa Mitaka, Tokyo, 181-8611 Japan

## Correspondence

Out-of-hospital cardiac arrest (OHCA) remains a major cause of mortality and morbidity worldwide. Moreover, families of OHCA patients are often confronted with a cascade of overlapping stressors including witnessing the event, participating in resuscitation as a bystander, making critical decisions in the intensive care unit (ICU), and assuming caregiving responsibilities. This traumatic sequence of events increases the risk of psychological symptoms, such as anxiety, depression, and post-traumatic stress disorder (PTSD) in family members [1]. Despite the recognized importance of providing psychological support to families of OHCA patients, evidence regarding their psychological symptoms and their trajectories remains limited. Therefore, the one-year trajectory of psychological symptoms in families of OHCA patients who were admitted to the ICU is presented.

From September 1, 2022, to March 31, 2024 at our hospital, one key decision-making family member per patient was enrolled if the ICU stay lasted ≥ 72 h. Exclusion criteria were as follows: patients and family members were under 18 years of age, absence of a key decision-maker for their care, and ICU stay was expected to be less than 72 hours.

After informed consent was obtained, questionnaires were sent to participants by mail 1 month, 3 months, and 1 year after hospital discharge. Anxiety and depression were assessed using the Hospital Anxiety and Depression Scale (HADS), PTSD with the Impact of Event Scale-Revised (IES-R), and resilience with the Connor-Davidson Resilience Scale-25 (CD-RISC-25). Scores ≥ 8 for the anxiety and depression components of the HADS survey were considered indicative of anxiety and depression, respectively. An average IES-R score ≥ 1.6 was considered indicative of PTSD.

**Fig. 1:**
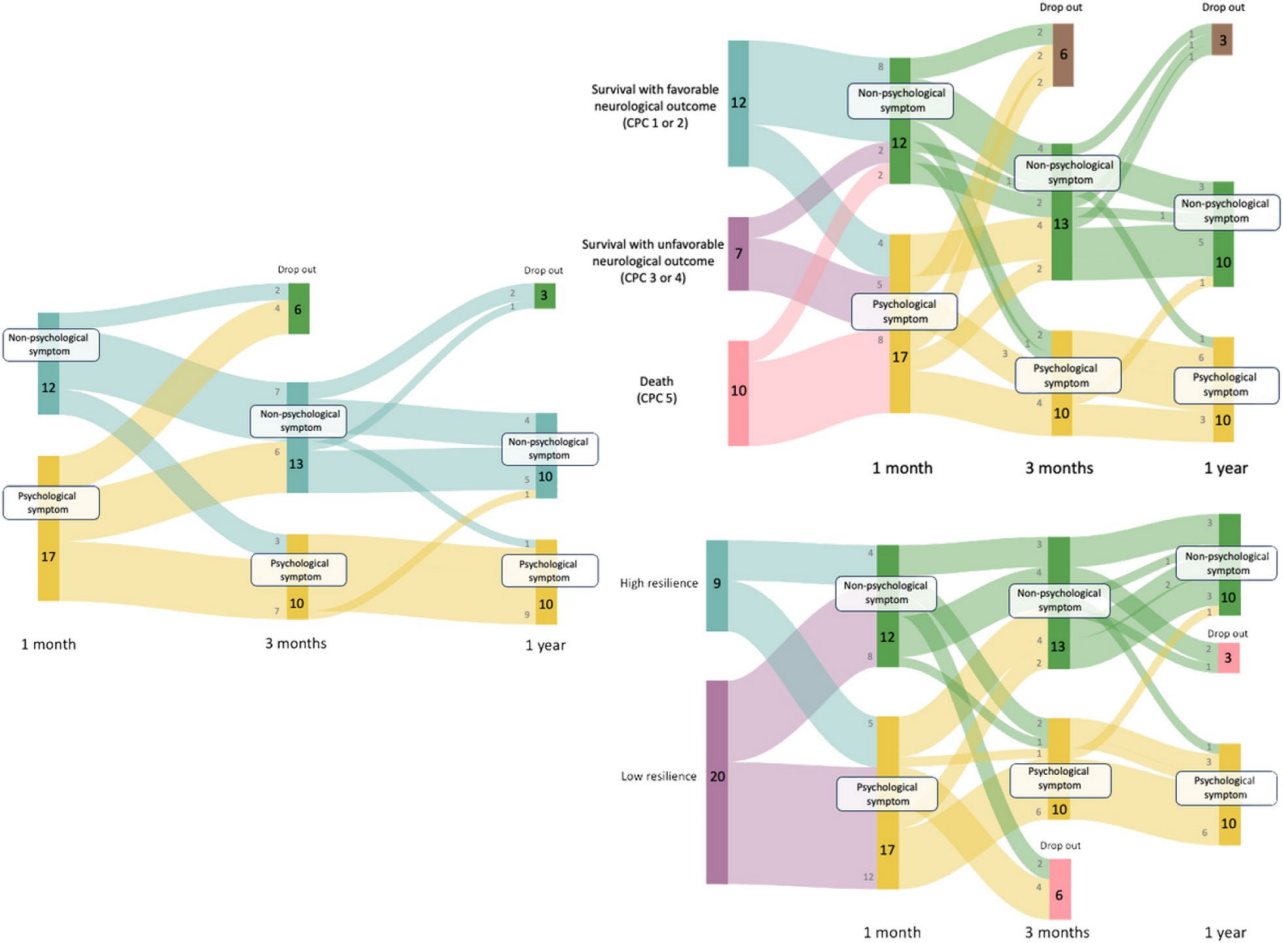
One-year trajectory of psychological symptoms in families of OHCA patients, and their associations with patient neurological outcomes and family resilience

Of 41 eligible families, 29 (71%) responded (Supplemental Fig. 1). The median age was 54 [interquartile range (IQR) 47–64] years, 7 (24.1%) were male (Supplemental Table 1). Of them, 17 (59%) had psychological symptoms one month after hospital discharge, and 10 (34.5%) had psychological symptoms one year later (Supplemental Figs. 1,2). Of families with psychological symptoms one month after hospital discharge, all (4/4) families of survivors with favorable neurological outcomes showed improvement three months later, whereas none of the families of survivors with unfavorable neurological outcomes showed improvement. In addition, all (5/5) resilient families showed improvement in their symptoms three months or one year later (Fig. 1).

Our findings have two important implications. First, most changes in symptoms occurred within the first three months, which suggest that some families of OHCA patients followed a natural trajectory of recovery within three months after hospital discharge. Similar trajectories have been reported in families of patients with other critical illnesses, which showed that most individuals recovered spontaneously within the first few months after a traumatic event, with only a minority developing long-term psychological disorders [2]. Previous studies of psychological symptoms of OHCA patients’ families have emphasized the high prevalence of such symptoms and the need for post-discharge follow-up systems [1, 3]. In contrast, our findings suggest that post-discharge support focused on families unlikely to recover spontaneously would be more effective. Therefore, future studies should explore whether families who exceed screening cutoffs three months after hospital discharge actually require mental health interventions, since whether all families with psychological symptoms at this time point should receive interventions remains unclear based on the present data. Clarifying these aspects will be essential for establishing recommendations on the optimal timing of psychological screening and appropriate intervention for families of OHCA patients.

Second, our findings suggest a novel hypothesis that patients’ neurological outcomes and family resilience may influence whether early psychological symptoms of families following ICU discharge resolve or persist over time. A previous study showed that caregivers of OHCA survivors with cognitive or functional impairments frequently experience sustained emotional burden, often due to the need to decrease employment or personal activities to accommodate caregiving responsibilities [4]. Furthermore, a previous qualitative study showed that family caregivers of OHCA survivors frequently experienced emotional conflicts, ongoing fear, and social isolation, while also feeling that their efforts were inadequately recognized by healthcare providers [5]. These emotional needs may contribute to the chronicity of psychological distress. In addition, the present finding that all families with high resilience at one month after discharge recovered within one year suggested that implementing resilience-enhancing interventions in the early post-discharge period may help support psychological recovery for families of OHCA patients. To confirm these hypotheses, further large-scale observational studies using appropriate statistical methods are needed.

In conclusion, approximately 30% of families of OHCA patients continued to experience psychological symptoms one year after hospital discharge. The psychological symptoms were mostly observed to resolve in families with high resilience and in those of survivors with favorable neurological outcomes.

## Supplementary Information

Supplementary Material 1: Flowchart of study participant selection. OHCA, out-of-hospital cardiac arrest; ICU, intensive care unit.

Supplementary Material 2: Occurrence of psychological symptoms one month after hospital discharge. The overlap of the circles represents the co-occurrence of the components. PTSD, post-traumatic stress disorder.

Supplementary Material 3.

## Data Availability

The datasets used and analyzed during the current research project are available from the corresponding author on reasonable request.

## Abbreviations

OHCA: Out-of-hospital cardiac arrest
ICU: Intensive care unit
PTSD: Post-traumatic stress disorder
HADS: Hospital anxiety and depression scale
IES-R: Impact of event scale-revised
CD-RISC-25: Connor-davidson resilience scale-25
IQR: interquartile range
