# Supplementary figures and images for: One-year trajectory of psychological symptoms in families of out-of-hospital cardiac arrest patients
Source: Crit Care. 2025 Oct 24;29:394. doi: 10.1186/s13054-025-05643-w (PMC12551261; doi:10.1186/s13054-025-05643-w)

Supplemental Fig. 1

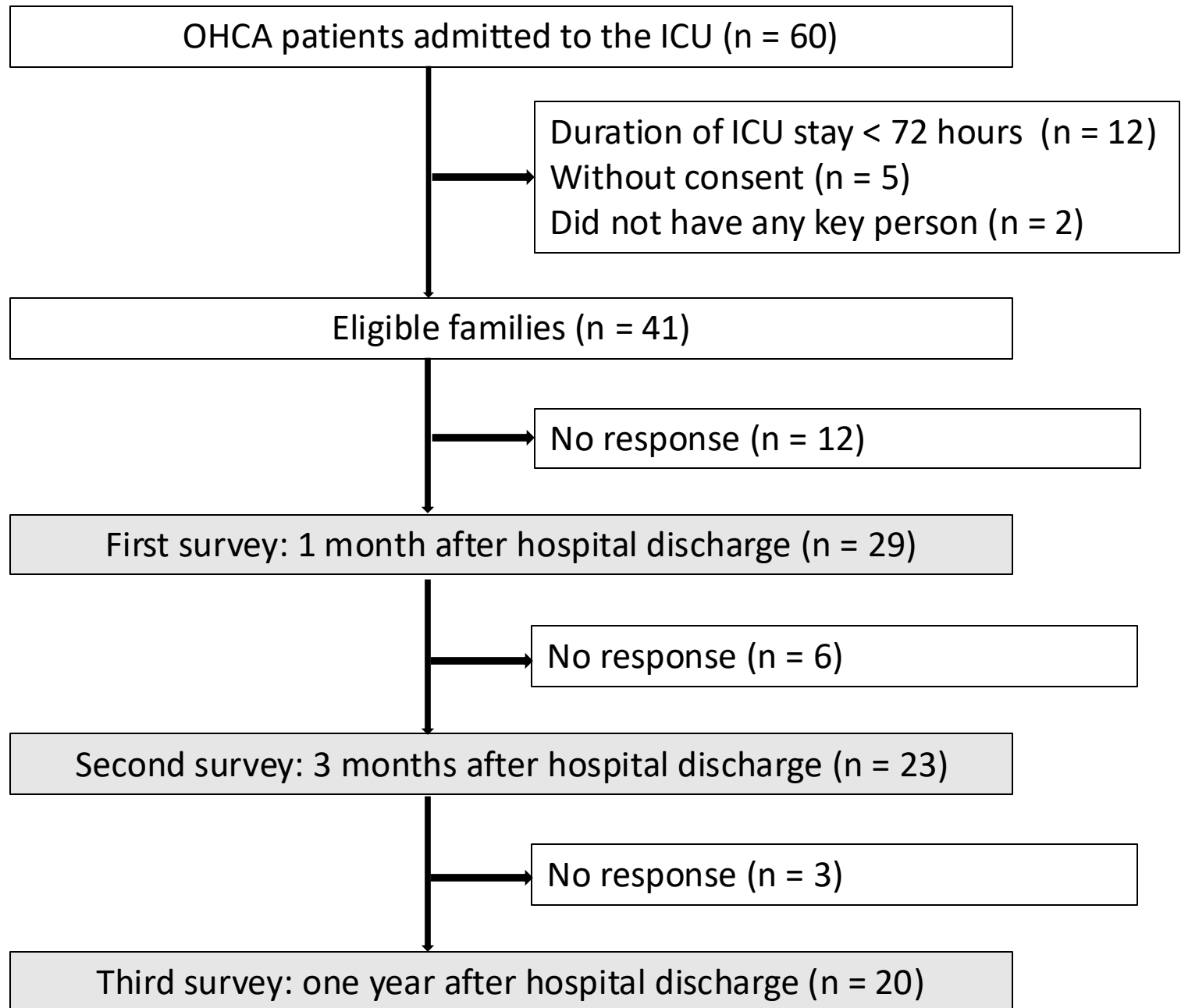

Supplement: Supplementary file 1 — Supplementary Material 1: Flowchart of study participant selection. OHCA, out-of-hospital cardiac arrest; ICU, intensive care unit. [file 13054_2025_5643_MOESM1_ESM.pdf]

Supplemental Fig. 2

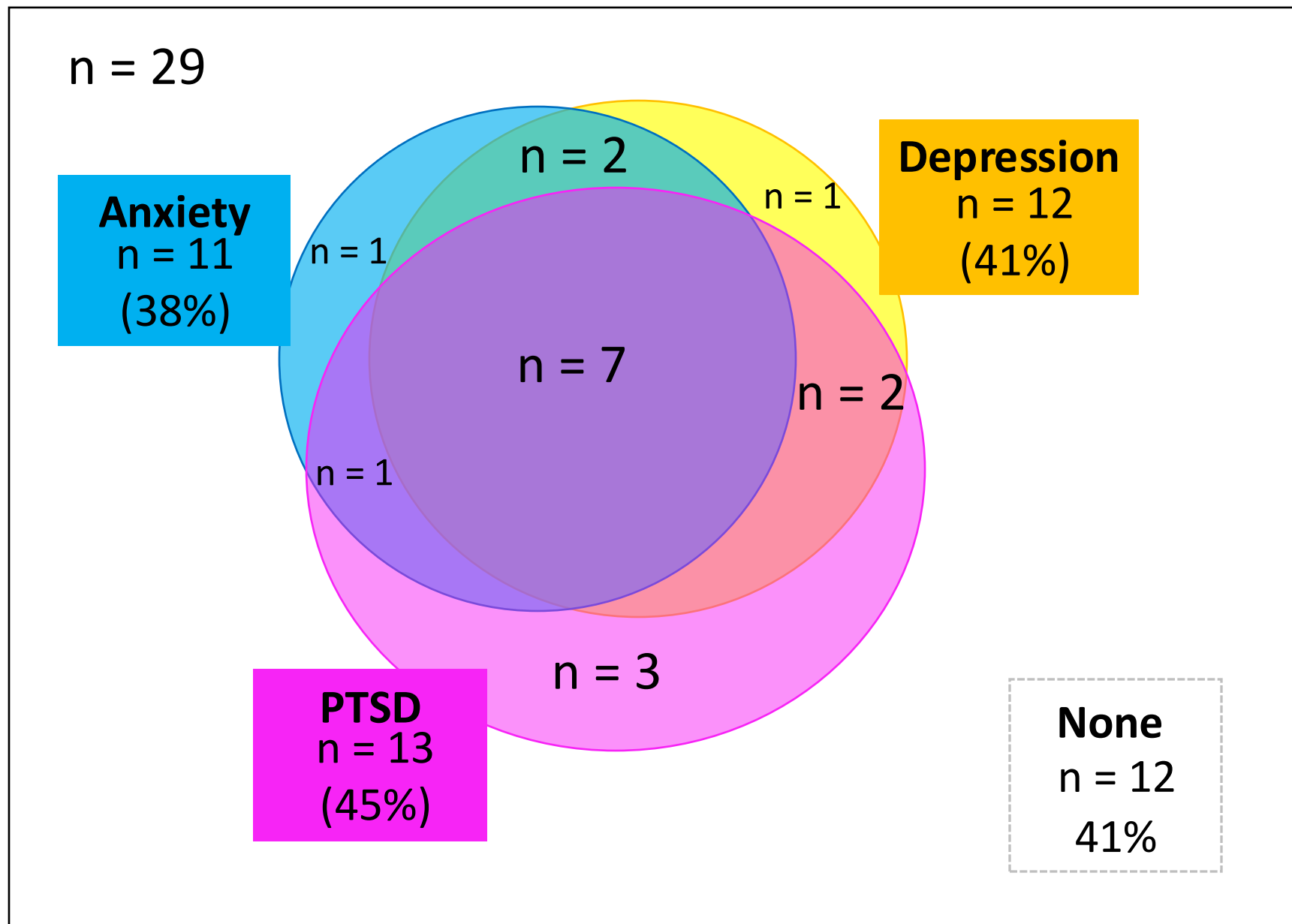

Supplement: Supplementary file 2 — Supplementary Material 2: Occurrence of psychological symptoms one month after hospital discharge. The overlap of the circles represents the co-occurrence of the components. PTSD, post-traumatic stress disorder. [file 13054_2025_5643_MOESM2_ESM.pdf]
