# Supplementary material for: One-year trajectory of psychological symptoms in families of out-of-hospital cardiac arrest patients
Source: Crit Care. 2025 Oct 24;29:394. doi: 10.1186/s13054-025-05643-w (PMC12551261; doi:10.1186/s13054-025-05643-w)
Supplement: Supplementary file 3 — Supplementary Material 3. [file 13054_2025_5643_MOESM3_ESM.docx]

**Supplemental Table 1. Participants’ baseline characteristics**

| **Variable** | **Total**  **(n = 29)** |
| --- | --- |
| **Patients** |  |
| Age (y), median [IQR] | 65 [48-75] |
| Sex (male), n (%) | 21 (75.0) |
| Length of hospital stay (days), median [IQR] | 24 [13-32] |
| Duration of ICU stay (days), median [IQR] | 7 [5-16] |
| Outcome at hospital discharge  Survival with favorable neurological outcome (CPC 1 or 2), n (%)  Survival with unfavorable neurological outcome (CPC 3 or 4), n (%)  Death (CPC 5), n (%) | 12 (41.4)  7 (24.1)  10 (34.5) |
| **Family members** |  |
| Age (y), median [IQR] | 54 [47-64] |
| Sex (male), n (%) | 7 (24.1) |
| Relationship with the patient  Spouse, n (%)  Child or parent, n (%)  Other family member, n (%) | 12 (41.4)  11 (37.9)  6 (20.7) |
| Highest level of education  High school or less, n (%)  University, n (%) | 16 (55.2)  13 (44.8) |
| Employed part or full-time, n (%) | 23 (79.3) |
| History of psychological disorder (Yes), n (%) | 3 (10.3) |
| FS-ICU score (total score), median [IQR] | 60 [55-66.5] |
| CD-RISC-25 score (total score), median [IQR] | 53 [46.5-71.5] |

Data are presented as medians [interquartile range] for continuous variables and as *N* (percentage) for categorical variables.

IQR = interquartile range; ICU = intensive care unit; CPC= Cerebral Performance Category; FS-ICU = Family Satisfaction in the ICU; CD-RISC-25 = Connor-Davidson Resilience Scale-25
